# Vascularized Tumor‐on‐a‐Chip Model as a Platform for Studying Tumor‐Microenvironment‐Drug Interaction

**DOI:** 10.1002/mabi.202500240

**Published:** 2025-07-22

**Authors:** Hyelim Kim, Seung‐Woo Cho, Hong Nam Kim

**Affiliations:** ^1^ Department of Biotechnology Yonsei University Seoul Republic of Korea; ^2^ Brain Science Institute Korea Institute of Science and Technology (KIST) Seoul Republic of Korea; ^3^ Center For Nanomedicine Institute for Basic Science (IBS) Yonsei University Seoul Republic of Korea; ^4^ Division of Bio‐Medical Science & Technology KIST School Korea University of Science and Technology (UST) Seoul Republic of Korea; ^5^ School of Mechanical Engineering Yonsei University Seoul Republic of Korea; ^6^ Yonsei‐KIST Convergence Research Institute Yonsei University Seoul Republic of Korea

**Keywords:** drug delivery, microphysiological system, tumor microenvironment, tumor‐on‐a‐chip

## Abstract

As cancer‐targeting technologies advance, robust platforms for evaluating drug delivery systems (DDS) under pathomimetic conditions are critically needed. Traditional models inadequately mimic human tumor microenvironment (TME) complexity due to interspecies variance, structural simplification, and static perfusion. Vascularized tumor‐on‐a‐chip systems address these gaps by integrating perfusable vasculature with tumor‐stroma dynamics in microfluidic environments, enabling dynamic 3D evaluation of drug transport kinetics and therapeutic efficacy. These advances significantly enhance preclinical‐to‐clinical translatability, though challenges remain in achieving long‐term vascular stability and multi‐tissue integration under physiological flow conditions. Herein, we summarize recent progress in vascularized tumor‐on‐a‐chip technologies for assessing DDS performance and TME interactions. Finally, opportunities for precision oncology and integrative organ‐level modeling are highlighted, underscoring the transformative potential of these platforms in next‐generation cancer research.

## Graphical abstract

Vascularized tumor‐on‐a‐chip platforms replicate human tumor microenvironments by integrating perfusable vessels and stromal elements in microfluidic systems. These models enable dynamic assessment of drug delivery and therapeutic response under physiologically relevant flow, offering enhanced predictive value for cancer treatment. This review highlights recent advances and future opportunities in precision oncology using such engineered systems.

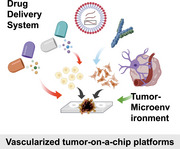

## 1 Introduction

Cancer remains a leading cause of mortality worldwide [1], underscoring the urgent need for innovative therapeutic strategies. According to the World Health Organization (WHO), cancer accounts for approximately 16% of global deaths, and the number of cancer cases is projected to rise by 52.6% by 2040 [2]. These alarming statistics highlight the growing burden of cancer and the importance of developing more effective and predictive platforms for cancer research and treatment. In response to these challenges, personalized cancer research focuses on tailoring treatments to individual tumor characteristics, yet it faces significant challenges such as tumor heterogeneity and the complexity of the tumor microenvironment (TME) [3, 4, 5, 6]. One critical aspect is the development of vascularized cancer models that replicate the dynamic interactions between tumors and their vasculature [7]. These models are essential for studying tumor growth, metastasis, and therapeutic responses since vascularization is a hallmark of cancer progression [8, 9, 10]. Angiogenesis enables tumors to grow beyond a certain size by providing nutrients and removing waste, making it a vital target for therapeutic interventions [11]. However, current preclinical models often fail to accurately mimic these processes, highlighting the need for advanced platforms that bridge the gap between in vitro simplicity and in vivo complexity.

Traditional in vitro models, such as 2D cell cultures and spheroids, have limitations in capturing the complexity of the TME, particularly its vascular components and the dynamic interactions between different cell types and the extracellular matrix [12, 13]. These limitations hinder their ability to accurately mimic tumor biology and predict drug responses. On the other hand, in vivo animal models provide a more realistic representation of human tumors but are constrained by interspecies differences, ethical concerns, and logistical challenges [14, 15]. The high failure rate of drugs transitioning from preclinical studies to clinical trials highlights the urgent need for more physiologically relevant models that bridge the gap between the simplicity of in vitro systems and the complexity of in vivo conditions [16, 17, 18, 19]. Developing advanced cancer models that better replicate these intricate interactions is essential for improving our understanding of tumor biology and enhancing drug development efforts [20, 21].

Despite significant progress in the development of 3D microfluidic chip models, several critical limitations remain that restrict their ability to accurately replicate complex biological systems. Many existing models primarily rely on single‐cell 3D cultures, which do not account for the interactions between multiple cell types, an essential aspect of mimicking in vivo conditions [22]. Furthermore, these models often lack mechanisms for precise control of fluid flow dynamics, which are crucial for recreating physiological microenvironments and nutrient transport [23, 24]. While drug treatments have been applied in some platforms, systematic evaluations of anticancer effects, such as drug efficacy and resistance, are frequently overlooked. Additionally, the absence of diverse analytical methods for anticancer assessment further limits their applicability in advanced drug screening and personalized medicine.

**FIGURE 1:**
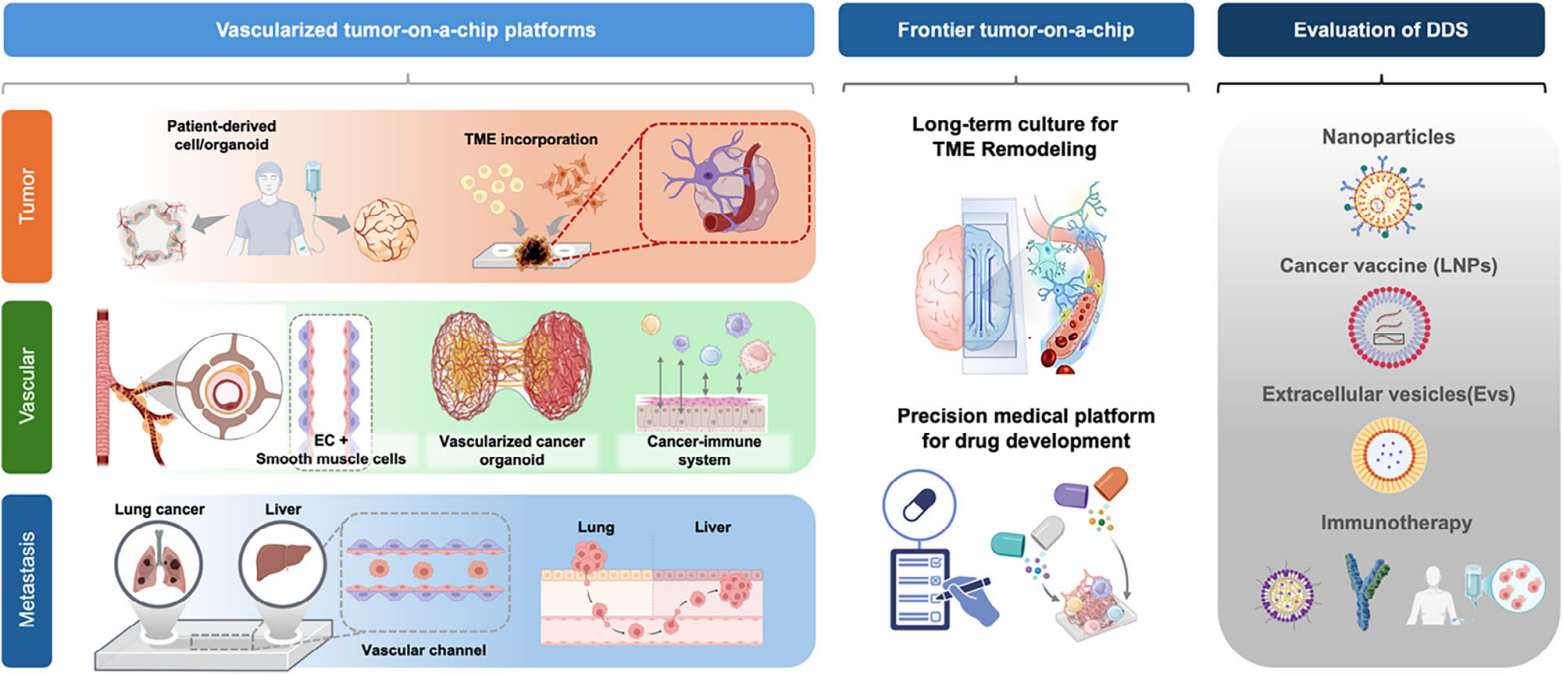
Schematic diagram of vascularized tumor‐on‐a‐chip platform. Moving beyond the inherent limitations of conventional 2D culture systems and reductionist tumor models, this microengineered tumor‐on‐a‐chip platform enables the reconstruction of a physiologically relevant tumor microenvironment. Through the integration of patient‐derived cells with stromal, endothelial, and immune cell populations, it establishes perfusable vasculature and recapitulates the architectural and cellular complexity of native human tissues. The incorporation of immune components facilitates the investigation of metastatic cascades and tumor–immune dynamics. This humanized vascularized model offers a translationally relevant tool for preclinical drug evaluation and precision oncology applications.

This review explores recent advancements in microfluidic chip technologies designed to overcome the limitations of traditional cancer models (Table 1). Microfluidic platforms enable precise control over fluid dynamics and cellular microenvironments at the microscale, offering unprecedented opportunities for tumor microenvironment modeling. The review will discuss various vascularized microfluidic chip designs and applications of drugs in cancer research, with a focus on clinical drug delivery, immunotherapy, and emerging applications such as tumor–immune cell co‐culture and organ‐specific barriers like the blood–brain barrier. These advances underscore the potential of tumor‐on‐a‐chip platforms to bridge the gap between preclinical modeling and personalized therapeutic strategies in precision oncology (Figure 1).

**TABLE 1:** Categorization of vascularized tumor‐on‐a‐chip platforms based on cell types and drug applications.

Category	Cell Types	Main Strategy	Drugs / Compounds	Findings	Representative Studies
Vascularized tumor‐on‐a‐chip model	Tumor cells + Endothelial cells + Fibroblasts	Perfusion‐enabled evaluation of drug transport	Paclitaxel, Doxorubicin, Trastuzumab	Drug‐dependent penetration and efficacy	[36, 40, 75]
Anti‐angiogenetic drug evaluation	Tumor spheroid + Endothelial cells	Assessment of VEGF inhibitor	Apatinib, Bevacizumab, Ramucirumab	Suppressed angiogenesis and integrity	[41, 42, 43]
Drug delivery system	Tumor spheroid + Endothelial cells	Targeted nanoparticle delivery	Nanoparticles, Liposomes + Anti‐tumor drugs	Accumulation under vascular conditions	[64, 66, 67, 68]
GBM‐on‐a‐chip model	Tumor spheroid + Brain Endothelial cells + Astrocyte + Pericyte	Penetration of targeted drugs across the BBB in GBM models	Mannitol, Doxorubicin	BBB opening and tumor drug accumulation	[71, 72]
BBB target delivery	Brain Endothelial cells + Astrocyte + Pericyte	Selective drug permeability	Caffeine, Cimetidine, Doxorubicin, Resveratrol, Osthole, Quercetin, Paeoniflorin, Wogonin, Matrine	Herbal compound BBB transport	[74]
Immune checkpoint drug	Tumor spheroid + Endothelial cells	PD‐1 Blockade & Immune Access	PD‐1/PD‐L1 inhibitor	Enhanced T cell cytotoxicity	[98, 99]
Immune cell delivery	Tumor cells + Endothelial cells	Tumor–Immune Interaction and Immunotherapy	T cells, CAR‐T cells	T cell transport and tumor infiltration	[100, 101, 102]
	Tumor cells + Endothelial cells		Myeloid cell (Monocyte, Macrophage)	Monocytes block extravasation	[103, 104]
	Tumor cells + Endothelial cells		NK cells	Rapid infiltration and tumor killing	[105, 106]

## 2 Consideration Factors for Engineering Tumor Vascularization

TME plays a crucial role in cancer progression, therapeutic resistance [25, 26], and metastatic potential [27]. Among its many components, tumor‐induced angiogenesis—the formation of new blood vessels from pre‐existing vasculature—is a hallmark of cancer and a key target for therapeutic intervention [28]. Abnormal tumor vasculature contributes to hypoxia, acidosis, and uneven drug distribution [29], which together promote tumor aggressiveness and limit therapeutic efficacy. As a result, modeling angiogenesis and its regulation within physiologically relevant microenvironments has become a central focus in cancer research [30]. Emerging microfluidic tumor‐on‐a‐chip platforms now offer the means to recapitulate the complexity of TME‐driven angiogenic dynamics in controlled, reproducible settings, enabling both mechanistic insight and therapeutic testing.

Recent tumor‐on‐a‐chip models have increasingly incorporated stromal elements such as fibroblasts to more accurately replicate the complex architecture of the tumor microenvironment. In particular, co‐culturing tumor cells with endothelial cells and fibroblasts within three‐dimensional extracellular matrix environments has been shown to enhance vascular self‐assembly, facilitating the formation of perfusable microvessels with physiologically relevant barrier properties [31]. In parallel, polydimethyl siloxane (PDMS)‐based microfluidic platforms with self‐assembled vascular lumens have enabled direct visualization of junctional proteins such as zonula occludens‐1 (ZO‐1) and vascular endothelial (VE)‐cadherin, along with quantitative assessment of vascular permeability using fluorescein isothiocyanate (FITC)‐dextran assays [32]. These systems have also proven especially useful for delineating how tumor‐derived biochemical signals—such as vascular endothelial growth factor (VEGF) secretion and extracellular matrix remodeling—interact with stromal cell‐driven organization to regulate angiogenic remodeling and compromise vascular integrity.

**FIGURE 2:**
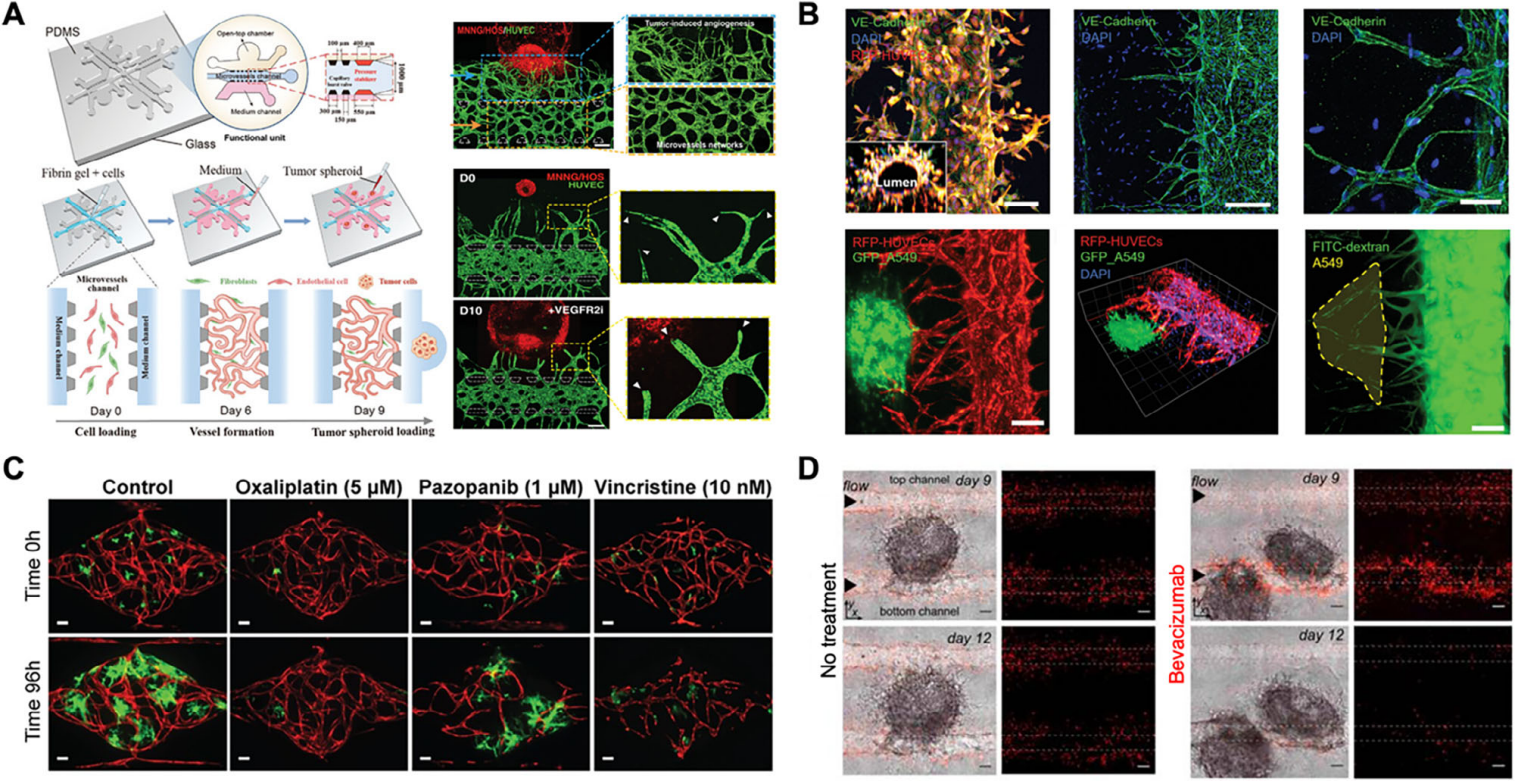
Engineering strategies for vascularized tumor‐on‐a‐chip platforms. (A) A patient‐derived tumor organoid (PDTO)‐on‐a‐chip platform incorporates a self‐assembled vascular network within a modular system to model tumor–vessel interactions. The device supports hierarchical microvasculature formation, PDTO invasion along vessels, and heterogeneous responses to VEGFR2 inhibitors, such as apatinib. Reproduced with permission [41]. Copyright 2025, Wiley‐VCH GmbH, Du *et al.* (B) A vascularized tumor‐on‐a‐chip model using A549 cells enabled evaluation of drug delivery, immune cell infiltration, and vessel occlusion by liquid metal nanoparticles under flow‐directed angiogenesis. Reproduced from the terms of the CC‐BY 3.0 license [33]. Copyright 2022, Kim *et al.* (C) A tumor‐on‐a‐chip model mimicking the vascularized tumor microenvironment was developed using co‐cultures of human tumor and stromal cells in 3D ECM with perfusable microvessels. It enabled evaluation of tumor growth and drug response in colorectal and breast cancer using agents like pazopanib, apatinib, and oxaliplatin. Adapted under the terms of the CC‐BY 4.0 International license [49]. Copyright 2016, Sobrino *et al.* (D) A microfluidic 3D tumor‐vascular model enabled real‐time observation of tumor‐induced angiogenesis and demonstrated the antiangiogenic efficacy of bevacizumab. Adapted under the terms of the CC‐BY 4.0 International license [40]. Copyright 2025, Skubal *et al.*

Moreover, tumor‐on‐a‐chip systems employing gravity‐driven flow across hydrogel chambers have effectively simulated interstitial pressure gradients, which promoted directional sprouting and capillary alignment. These gradients also enhanced endothelial survival and lumen maintenance, closely mimicking the mechanical cues that govern vascular morphogenesis in vivo (Figure 2B) [33, 34, 35]. Further advancing the fidelity of vascularized delivery platforms, the Channel‐Assembling Tumor Microenvironment‐on‐a‐Chip (CATOC) system introduced a modular approach that separates vascular and tumor compartments while preserving directional drug transport. This PDMS‐based chip allowed trans‐endothelial administration of chemotherapeutic agents such as doxorubicin and trastuzumab from the vascular to the tumor side. When applied to breast cancer spheroids derived from BT474 and MCF7 cell lines, the model recapitulated subtype‐specific drug responses, confirming the importance of vascular transport in modulating therapeutic outcomes. In comparison to scaffold‐free systems, the CATOC platform enabled more consistent evaluation of drug penetration, uptake, and cytotoxicity across multiple tumor types, supporting its potential for standardized drug screening and physiologically relevant delivery assessment [36]. In addition to stromal and vascular elements, recent efforts in tumor‐on‐a‐chip engineering have begun incorporating immune cells such as T lymphocytes, macrophages, and natural killer (NK) cells to more accurately reflect the immunological dimension of the TME [37]. These immune cells can influence angiogenic remodeling through cytokine secretion (e.g., VEGF, IL‐1, IL‐6, TNF‐α) and direct interaction with endothelial cells, thereby modulating vascular integrity and permeability [38]. Cancer‐associated fibroblasts (CAFs), beyond their structural roles, also contribute to immune suppression by altering extracellular matrix composition and secreting factors like TGF‐β and IL‐1, which can inhibit immune cell infiltration and function [39]. Integrating these immune components into vascularized tumor models strengthens the translational relevance of the system and lays the groundwork for evaluating immunotherapeutic responses, as described in Section 3.3.

## 3 Application of Vascularized Tumor‐on‐a‐Chip Platform

Extending from these findings, several studies have demonstrated the utility of these systems in evaluating drug efficacy and delivery kinetics in vascularized tumor contexts. This section highlights key applications of these platforms in evaluating the performance of anticancer drugs, drug delivery systems (DDS), and immunotherapeutic strategies.

### 3.1 Anti‐Angiogenic Drug Evaluation

This section focuses on the vasculature, as its inclusion represents a significant advancement over traditional tumor spheroid models. Microfluidic 3D tumor‐vascular model enabled co‐culture of cancer spheroids with endothelial networks to assess the impact of antiangiogenic therapy. Upon treatment with apatinib, bevacizumab, and ramucirumab, a clinically used VEGF inhibitor, the model revealed suppression of tumor‐induced neovascularization and disruption of vascular integrity, validating its effectiveness in recapitulating angiogenic modulation and vascular response in vitro (Figure 2A,D) [40, 41, 42, 43]. Similarly, angiogenesis‐modulating drugs, notably the γ‐secretase inhibitor DAPT (N‐[N‐(3,5‐difluorophenacetyl)‐L‐alanyl]‐S‐phenylglycine t‐butyl ester), which targets the Notch signaling pathway, were tested using a system based on spontaneous capillary flow‐driven 3D co‐culture patterning [44].

Interestingly, it was found that even media components such as sodium selenite—commonly used in serum‐free formulations—could compromise vascular integrity at low concentrations, underscoring the importance of media optimization in angiogenesis studies [45]. In parallel, evaluation of glioblastoma‐induced hypercoagulability demonstrated the anticoagulant efficacy of Rivaroxaban and a tissue factor‐blocking antibody using a cancer‐on‐a‐chip platform [46]. Additionally, comparative drug testing revealed that vessel stabilization and permeability outcomes varied depending on drug class and concentration. These findings highlight the platform's potential to screen candidate therapies for vascular normalization or inhibition under controlled and quantitative conditions.

In addition to drug screening, these platforms support the investigation of tumor‐associated vascular mechanisms by providing mechanistic insights into tumor‐driven vascular pathologies. The pancreatic ductal adenocarcinoma‐on‐a‐chip model revealed a unique endothelial ablation mechanism mediated by activin‐ALK7 signaling, whereby pancreatic tumor cells actively displaced adjacent endothelial cells to create hypovascular zones—a feature commonly observed in pancreatic ductal adenocarcinoma [47]. Similarly, glioblastoma (GBM)‐on‐a‐chip model with controlled oxygen gradients was used to study hypoxia‐adapted tumor responses. The tetralol compound (a highly selective T‐type calcium channel blocker) selectively induced apoptosis in hypoxic cores, suppressed hypoxia inducible factor 1‐α (HIF1‐α) expression, and inhibited late‐stage autophagy. The findings highlight its potential to target hypoxia‐driven mechanisms in GBM [48]. This ablation process was confirmed using both in vitro and in vivo models, demonstrating the translational relevance of chip‐based findings. In other studies, colorectal and breast cancer models displayed distinct angiogenic responses to multi‐targeted kinase inhibitors such as cabozantinib and linifanib, depending on tumor type and metabolic profile (Figure 2C) [49, 50].

Together, these systems offer a high‐fidelity representation of the spatial and temporal dynamics of angiogenesis in diverse tumor settings, advancing our understanding of tumor–vasculature interactions and enabling the rational design of antiangiogenic therapies.

### 3.2 Evaluation of Drug Delivery System (DDS) Efficiency

Efficient drug delivery in cancer therapy remains a significant challenge due to the abnormal and dysfunctional nature of tumor vasculature in solid tumors [51], which is characterized by irregular architecture, elevated interstitial fluid pressure, and uneven perfusion gradients [52, 53]. These factors severely limit drug penetration and reduce therapeutic efficacy. To address these challenges, microfluidic tumor‐on‐a‐chip platforms with perfusable vasculature have emerged as powerful tools for studying drug transport under physiologically relevant conditions [54]. These systems replicate dynamic flow [55], vascular barrier integrity [56, 57], and tissue‐specific characteristics, enabling detailed analysis of pharmacokinetics and pharmacodynamics [58, 59, 60]. Additionally, their ability to provide high‐resolution spatiotemporal visualization of drug movement makes them ideal for optimizing delivery strategies [61, 62], such as nanoparticle functionalization or vascular normalization, and refining therapeutic regimens to improve treatment outcomes [63].

**FIGURE 3:**
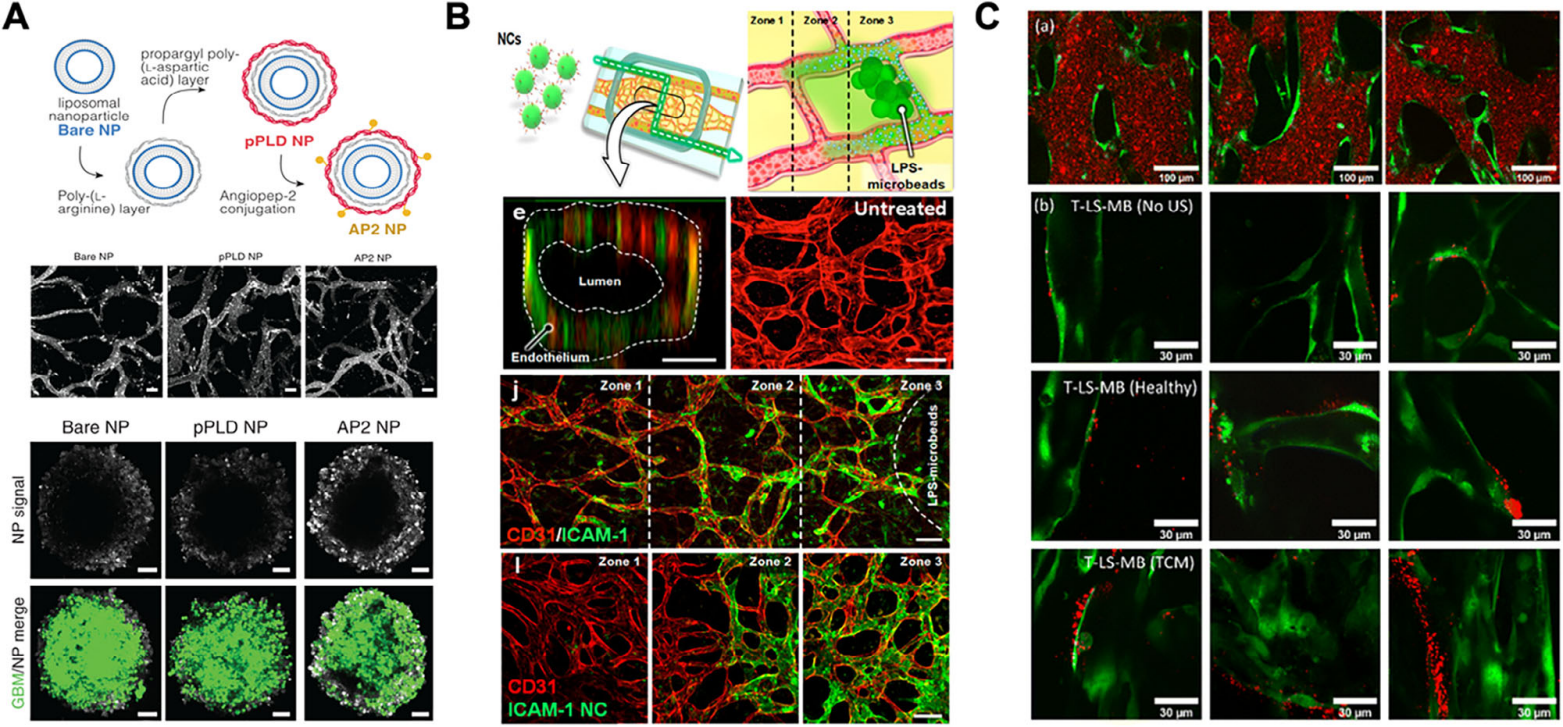
Vascularized tumor‐on‐a‐chip platforms for drug delivery. (A) A GBM‐on‐a‐chip system integrating self‐assembled vasculature with tumor spheroids replicates BBB characteristics using human endothelial cells, astrocytes, and pericytes. Functionalized nanoparticles encapsulating cisplatin demonstrated improved BBB penetration and tumor accumulation compared to free drug delivery, highlighting the platform's translational potential for CNS‐targeted therapy. Adapted under the terms of the CC‐BY 4.0 International license [73]. Copyright 2022, Straehla *et al.* (B) Azide‐functionalized dipalmitoylphosphatidylcholine (DPPC)‐cholesterol liposomes were tested in a 3D microfluidic model with engineered microvessels to assess intravascular delivery, tumor cell killing, and vascular toxicity under inflammatory conditions. The platform enabled evaluation of nanoengineered therapies but requires further refinement for long‐term and hierarchical vascular modeling. Reproduced with permission from Copyright 2019 American Chemical Society [67]. Paek *et al.* (C) A tumor‐associated vasculature model without tumor cells, co‐cultures of endothelial cells and fibroblasts in a microfluidic chip to study vascular targeting efficiency. Integrin αvβ3‐targeted liposomes combined with ultrasound‐mediated microbubbles showed significantly enhanced accumulation and drug delivery efficiency, offering a tumor‐mimicking, non‐tumor‐dependent screening strategy. Reproduced under the terms of the CC‐BY 3.0 license [64]. Copyright 2023, Bourn *et al.*

Nanoparticle and liposome‐based delivery systems have shown enhanced tumor specificity and reduced systemic toxicity in preclinical models, and their evaluation in vascularized microfluidic platforms has proven particularly informative. For example, αvβ3 integrin‐targeted liposomes exhibited significantly greater vascular adhesion and accumulation in engineered tumor vasculature compared to non‐targeted controls (Figure 3C) [64]. Additionally, using HUVECs and SKOV3 cells, the platform demonstrated in vivo‐relevant nanoparticle (NP) distribution patterns for polyethylene glycol (PEG)‐liposomes and PEG‐poly(lactic‐*co*‐glycolic) acid (PLGA) NPs [65]. Similarly, azide‐functionalized fluorescent liposomes demonstrated improved drug delivery efficiency in inflammatory vascular models, while hepatic metastasis‐on‐a‐chip systems confirmed the activation and efficacy of prodrugs in liver‐mimicking environments (Figure 3B) [66, 67]. Sex‐specific platforms incorporating triple‐negative breast cancer spheroids and endothelial cells revealed that phytochemicals like glyceollin exhibit distinct vascular effects based on biological sex, further supporting the relevance of microfluidic systems for personalized drug screening [68].

Blood–brain barrier (BBB)‐on‐a‐chip platforms have proven essential for modeling the selective and dynamic nature of central nervous system (CNS) drug transport, particularly in GBM treatment [69, 70]. Triculture models incorporating human brain microvascular endothelial cells, astrocytes, and pericytes successfully recreated key BBB characteristics, including tight junction formation and regulated permeability [71]. In a GBM‐on‐a‐chip model, researchers constructed perfusable cylindrical blood vessels by microneedle removal and co‐cultured them with T98G glioblastoma spheroids. The model incorporated a physiologically relevant triculture BBB composed of endothelial cells, astrocytes, and pericytes. Upon osmotic opening with D‐mannitol, BBB‐impermeable drugs such as vincristine and doxorubicin successfully penetrated the barrier and accumulated in the tumor, as verified through real‐time fluorescence imaging [72]. Another study employed a vascularized GBM‐on‐a‐chip model to test nanoparticle‐mediated cisplatin delivery; BBB‐targeted nanoparticles improved tumor penetration and therapeutic efficacy while recapitulating in vivo permeability patterns (Figure 3A) [73]. Meanwhile, a separate chip model was used to evaluate the BBB transport of six traditional Chinese medicine‐derived anti‐glioma compounds using high‐performance liquid chromatography‐ultraviolet, confirming selective permeability and differential drug accumulation profiles [74]. Together, these platforms underscore the importance of physiologically relevant BBB models in CNS drug development, enabling both mechanistic studies and high‐throughput screening of therapeutics under realistic barrier conditions.

In addition to testing formulations, vascularized chips also allow for evaluation of how tumor microenvironmental features such as vessel size, network density, and flow rates impact drug delivery. Hierarchical vascular chips with self‐assembled capillaries and perfusable lumens revealed that interconnected vessel networks promote better nutrient and drug diffusion, resulting in altered tumor growth and resistance dynamics [75, 76, 77]. These models also enabled the assessment of chronic drug exposure and long‐term vascular remodeling, factors that are often missed in short‐term in vitro assays.

Building on this potential, many studies have begun to extend culture durations to better capture delayed responses and sustained tissue remodeling [78]. While these efforts highlight the growing interest in long‐term modeling, recent studies reflect a range of approaches—from several days to multiple weeks—and are increasingly exploring how key functions such as barrier integrity and cellular phenotype can be sustained throughout extended culture periods [79]. This variability underscores the need for more consistent evaluation standards to fully realize the benefits of tumor‐on‐a‐chip platforms. With integrated imaging and computational modeling, these platforms offer predictive insights into treatment outcomes and provide a bridge between conventional preclinical models and clinical trials [66, 67, 73, 80].

### 3.3 Investigating Tumor–Immune Interaction and Evaluating Cancer Immunotherapy

Recent advances in cancer immunotherapy have reshaped treatment paradigms, yet clinical outcomes remain limited by the immunosuppressive TME [71, 81, 82]. The TME's complexity—encompassing stromal networks, vascular abnormalities, and biochemical signaling—creates dynamic barriers to immune cell infiltration and function [83, 84]. Conventional models inadequately mimic these interactions, failing to capture spatial, mechanical, and physiological TME features [85]. Vascularized tumor‐on‐a‐chip platforms address this by combining 3D tumor models, functional vasculature, and stromal‐immune components in a tunable microfluidic system [86]. These systems enable real‐time analysis of immune cell dynamics, drug delivery efficiency, and therapy‐induced microenvironmental changes [87, 88]. By reconstructing TME‐driven immune evasion mechanisms [89], they provide insights into resistance pathways across cancer subtypes [90]. Furthermore, they serve as scalable platforms for testing combinatorial therapies, such as immune‐modulating agents with targeted drugs [91, 92, 93, 94, 95]. Integration of patient‐derived cells allows personalized evaluation of therapeutic efficacy and TME reprogramming potential [96, 97]. These advancements position tumor‐on‐a‐chip systems as transformative tools for bridging preclinical discovery and clinical translation. Ultimately, they offer a pathway to optimize precision immunotherapy strategies and overcome microenvironment‐mediated treatment barriers.

**FIGURE 4:**
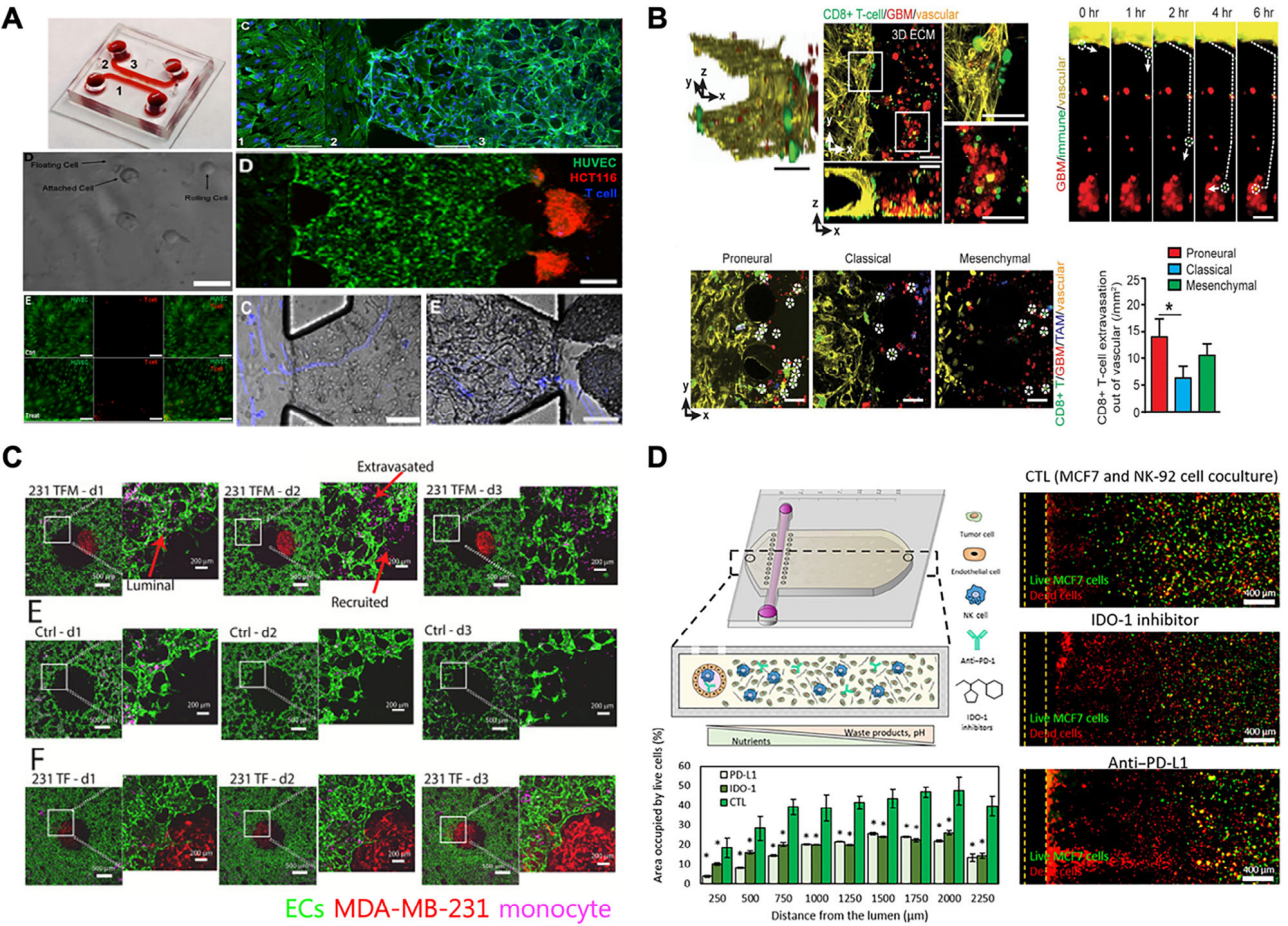
Immune–tumor interactions and modulation within vascularized tumor‐on‐a‐chip platforms. (A) A vascularized tumor‐on‐a‐chip model integrates endothelial‐lined vessels and tumor spheroids to visualize T cell trafficking and tumor infiltration under dynamic flow. PD‐1 checkpoint blockade enhances T cell cytotoxicity, and the endothelial glycocalyx regulates immune cell entry. Adapted under the terms of the CC‐BY 4.0 International license [98]. Copyright 2024, Zhao *et al.* (B) A GBM‐on‐a‐chip model incorporating tumor cells, CD8+ T cells, and CD163+ TAMs was used to test nivolumab (PD‐1 inhibitor) alone and with BLZ945 (CSF‐1R inhibitor), demonstrating effective depletion of M2‐like TAMs and enhanced T cell cytotoxicity in mesenchymal GBM. Adapted under the terms of the CC‐BY 4.0 International license [100]. Copyright 2020, Cui *et al.* (C) A vascularized co‐culture system with tumor spheroids and circulating monocytes models TAM polarization. A multispecific antibody targeting CSF‐1R, CCR2, and TGF‐β suppresses monocyte infiltration and reprograms macrophages toward an anti‐tumor (M1‐like) phenotype in breast and lung cancer models. Adapted with permission. Copyright 2025, Elsevier [103]. Nguyen *et al.* (D) A hypoxia‐mimicking tumor‐on‐a‐chip with dense MCF7 spheroids simulates NK cell exhaustion under metabolic stress. NK‐92 cells lose cytotoxicity over time but can be reactivated by antibody–cytokine conjugates or immune checkpoint inhibitors, a phenomenon not observed in conventional 2D cultures. Adapted under the terms of the CC‐BY 4.0 International license [106]. Copyright 2021, Ayuso *et al.*

Another platform enabled real‐time imaging of T cell transport and tumor infiltration, demonstrating that programmed cell death protein 1 (PD‐1) blockade enhanced cytotoxicity and identified the endothelial glycocalyx as a regulator of immune access (Figure 4A) [98]. A lung tumor‐on‐a‐chip system incorporating autologous T cells and cancer‐associated fibroblasts showed that stromal elements significantly reduced anti‐PD‐1 efficacy via CXCL12‐mediated suppression of T cell cytotoxicity [99]. Similarly, GBM‐on‐a‐chip models revealed that mesenchymal glioblastoma niches expressed high levels of PD‐L1 and CSF1, attracting CD163+ M2 macrophages and suppressing CD8+ T cell infiltration. Combination therapy with PD‐1 inhibitors and CSF1R inhibitors restored cytotoxic T cell activity and increased tumor apoptosis, emphasizing the utility of these chips for evaluating combination immunotherapies (Figure 4B) [100]. In hepatocellular carcinoma models, monocyte‐derived suppression of TCR‐transduced T cells was observed via PD‐L1/PD‐1 signaling, with suppression limited to cells transduced by retroviral methods—not those transfected by mRNA electroporation—highlighting the relevance of delivery method in T cell immunogenicity [101]. To enhance immune cell access, another study introduced a sequential fibroblast integration strategy that promoted peripheral vascularization around tumor spheroids. This method, applied across kidney, lung, and ovarian cancer models, improved chimeric antigen receptor T‐cell (CAR‐T) cell infiltration and function, though its efficacy was reduced in non‐aggregating tumor types or in conditions where fibroblasts rapidly migrated inward, indicating the need for cell line‐specific optimization [102]. These systems highlight the need to consider both tumor‐intrinsic and extrinsic factors in immunotherapy design.

Immune‐on‐a‐chip platforms also offer insights into monocyte behavior and the impact of immune cell maturation state. A 3D co‐culture model of vascular networks and tumor spheroids was developed to study monocyte infiltration and tumor‐associated macrophage (TAM) polarization in breast and lung cancers. The platform tested a multispecific antibody targeting CSF‐1R, CCR2, and TGF‐β, showing significant suppression of monocyte recruitment and reprogramming of TAMs toward an M1‐like phenotype. Patient‐derived tumor models corroborated these findings. This study demonstrates the value of combinatorial targeting of immunosuppressive pathways in the tumor microenvironment (Figure 4C) [103]. In breast cancer models, undifferentiated monocytes migrating through vascularized microenvironments inhibited tumor cell extravasation via actomyosin‐dependent mechanisms, while differentiated macrophages lost this anti‐metastatic function [104]. Collectively, these findings demonstrate that vascularized immune‐chip platforms are not only effective tools for drug screening, but also mechanistic platforms to dissect immune signaling pathways and refine immunotherapy strategies under patient‐specific and tumor‐type‐specific conditions.

NK cell‐based immunotherapies are increasingly explored as a strategy to target solid tumors, yet understanding their behavior in the TME requires models that preserve tissue architecture and metabolic constraints. A 3D vascularized breast tumor‐on‐a‐chip platform was developed using MCF7 spheroids vascularized by endothelial cell channels to investigate NK cell infiltration and tumor cytotoxicity. NK‐92 and NK‐92.CD16V cells perfused through the vascular channels rapidly infiltrated tumor spheroids and effectively targeted both peripheral and central regions, outperforming antibody‐based therapies that were limited by diffusion [105]. In a complementary study, a hypoxia‐mimicking tumor‐on‐a‐chip model revealed that NK cells progressively lost cytotoxic function under nutrient‐deprived conditions, and this exhaustion persisted even after removal from the suppressive environment. Notably, immune checkpoint inhibitors such as anti‐PD‐1 and IDO‐1 were only effective in 3D chip settings, not in 2D culture, underscoring the importance of spatial and metabolic context in immune modeling [106]. (Figure 4D) Collectively, these models emphasize the importance of vascular context, metabolic environment, and stromal modulation in shaping immune cell performance within tumors.

## 4 Challenges and Prospects

Vascularized tumor‐on‐a‐chip platforms have shown great promise as in vitro models for evaluating DDS under conditions that closely resemble the human tumor microenvironment. By integrating perfusable vascular networks and enabling precise control over dynamic flow, tissue architecture, and drug exposure, these systems offer a more physiologically relevant alternative to traditional static cultures and even some in vivo models. However, while their value in bridging preclinical testing and clinical translation is evident, several limitations must be addressed before these platforms can be widely adopted for systematic and large‐scale therapeutic development.

One of the key challenges lies in the diversity and physiological relevance of the cellular components used. Most current models rely on a limited set of endothelial or tumor cell lines, which may not fully capture the heterogeneity seen in patient tumors. Incorporating a wider variety of cell types—including pericytes, cancer‐associated fibroblasts, and diverse immune cell populations—has been shown to significantly affect vascular formation, immune infiltration, and drug response [31, 32, 42, 103, 104]. This cellular diversity is especially important for accurately replicating the spatial and functional complexity of the tumor microenvironment, particularly in studies involving immune‐modulating therapies or microenvironment‐driven resistance mechanisms.

Another critical direction for future development is the integration of artificial intelligence (AI) and machine learning tools to process the large datasets generated by these microfluidic platforms. Several recent studies have employed high‐throughput tumor‐on‐a‐chip systems in combination with nanoparticle‐based DDS and computational modeling to analyze drug delivery efficiency and tumor response [66, 67, 68, 73]. These approaches enable multiparametric evaluation of pharmacokinetics, vascular remodeling, and cell viability, and may provide the foundation for predictive modeling in precision oncology.

The use of patient‐derived cells represents another major step toward clinical translation. Compared to immortalized cell lines, patient‐derived tumor organoids or primary tumor spheroids preserve interpatient heterogeneity and have demonstrated differential responses to antiangiogenic or cytotoxic agents in vascularized microfluidic environments [34, 41, 49, 50, 107, 108]. The combination of such patient‐derived constructs with perfusable vascular networks and real‐time imaging systems offers a robust framework for functional drug testing and individualized treatment optimization. However, ensuring long‐term viability and functional integration of these cells in microscale environments remains a technical hurdle [42, 45]. In addition, clinical application is often constrained by the time required to generate fully functional microphysiological constructs from patient‐derived samples [109]. While automation of imaging or fluid handling can streamline workflow, such methods cannot fully resolve the biological time needed for vascular network formation or organoid maturation. To address this issue, emerging strategies such as pre‐vascularized scaffolds, rapid maturation protocols, and modular tissue assembly are being explored to reduce turnaround time and support more timely decision‐making in personalized therapy [110].

In addition, the translation of tumor‐on‐a‐chip systems into scalable drug screening tools requires compatibility with high‐throughput formats. Modular designs and compartmentalized flow architectures have been applied to enable parallel testing of therapeutic conditions, allowing simultaneous comparison of different tumor subtypes or drug regimens within a single chip device [36, 46]. Automating such platforms and integrating them with robotic handling systems could significantly accelerate early‐stage therapeutic evaluation and phenotypic screening.

Advancing toward multi‐organ‐on‐a‐chip configurations will further extend the utility of these systems. Studies connecting tumor compartments with liver, lymphoid, or BBB modules have demonstrated the potential to analyze systemic effects, drug metabolism, and inter‐tissue signaling [33, 41, 73, 111]. However, achieving functional balance across different compartments requires careful medium formulation. For example, media optimized for one cell type may inhibit differentiation or function of another; in BBB models, using a mixture of human neural stem cells and endothelial cell media significantly improved astrocyte differentiation, underscoring the importance of media optimization in complex multi‐cell systems [72, 74, 112].

Another promising direction involves the integration of biosensors into tumor‐on‐a‐chip platforms for real‐time monitoring of barrier integrity, oxygen levels, drug diffusion, and tissue viability. This not only increases data resolution but also supports long‐term culture applications, which are crucial for studying chronic drug exposure or delayed therapeutic responses [45, 46]. Despite the advances in short‐term modeling, maintaining stable cellular phenotypes and functional tissues for extended durations remains a critical need in replicating chronic treatment environments.

In conclusion, vascularized tumor‐on‐a‐chip systems have evolved into powerful preclinical models, but their future impact will depend on further progress in five key areas: incorporation of diverse and physiologically relevant cell types, integration of AI and computational analysis, utilization of patient‐derived cells for personalized testing, application of high‐throughput and automation‐compatible designs, and development of multi‐organ systems to investigate systemic effects and complex drug responses. These directions collectively represent the next frontier in preclinical modeling, offering new opportunities for innovation in oncology drug development and personalized therapeutic strategies.

## Author Contributions

H.K. performed writing–review & editing, writing–original draft, investigation, and conceptualization. S.‐W.C. performed writing‐review & editing, supervision, and project administration. H.N.K. performed writing‐review & editing, conceptualization, supervision, and project administration.

## Conflicts of Interest

The authors declare no conflicts of interest.

## Data Availability

The data that support the findings of this study are available from the corresponding author upon reasonable request.
